# Dissemination and Transmission of the E1-226V Variant of Chikungunya Virus in *Aedes albopictus* Are Controlled at the Midgut Barrier Level

**DOI:** 10.1371/journal.pone.0057548

**Published:** 2013-02-21

**Authors:** Camilo Arias-Goeta, Laurence Mousson, François Rougeon, Anna-Bella Failloux

**Affiliations:** 1 Department of Virology, Arboviruses and Insect Vectors, Institut Pasteur, Paris, France; 2 Cellule Pasteur, Université Pierre et Marie Curie, Paris, France; 3 URA CNRS 2581, Institut Pasteur, Paris, France; University of Texas Medical Branch, United States of America

## Abstract

Emergence of arboviruses could result from their ability to exploit new environments, for example a new host. This ability is facilitated by the high mutation rate occurring during viral genome replication. The last emergence of chikungunya in the Indian Ocean region corroborates this statement since a single viral mutation at the position 226 on the E1 glycoprotein (E1-A226V) was associated with enhanced transmission by the mosquito *Aedes albopictus* in regions where the major mosquito vector, *Aedes aegypti*, is absent.

We used direct competition assays *in vivo* to dissect out the mechanisms underlying the selection of E1-226V by *Ae. albopictus*. When the original variant E1-226A and the newly emerged E1-226V were provided in the same blood-meal at equal titers to both species of mosquitoes, we found that the proportion of both variants was drastically different in the two mosquito species. Following ingestion of the infectious blood-meal, the E1-226V variant was preferentially selected in *Ae. albopictus*, whereas the E1-226A variant was sometimes favored in *Ae. aegypti*. Interestingly, when the two variants were introduced into the mosquitoes by intrathoracic inoculations, E1-226V was no longer favored for dissemination and transmission in *Ae. albopictus*, showing that the midgut barrier plays a key role in E1-226V selection.

This study sheds light on the role of the midgut barrier in the selection of novel arbovirus emerging variants. We also bring new insight into how the pre-existing variant E1-226V was selected among other viral variants including E1-226A. Indeed the E1-226V variant present at low levels in natural viral populations could rapidly emerge after being selected in *Ae. albopictus* at the midgut barrier level.

## Introduction

Chikungunya virus (CHIKV) is a re-emerging arthropod-borne virus (Togaviridae, *Alphavirus*) responsible for recent severe epidemics. The disease caused by CHIKV is characterized in humans by an acute illness with high fever, rash, headache, myalgia and incapacitating arthralgia [1]. Chikungunya (CHIK) is endemic in several countries in Africa, the Indian sub-continent and Southeast Asia. First isolated in 1952 in Tanzania, CHIKV is historically found in Africa where it circulates within enzootic cycles involving forest-dwelling mosquitoes and wild non-human primates [2], [3]. In Asia, where the first outbreak was reported in 1958 in Thailand, CHIKV appears to be maintained in a strictly urban cycle where inter-human transmission is mainly carried out by the mosquito *Aedes aegypti* and to a lesser extent, *Aedes albopictus* [4]. In 2004, CHIKV emerged in Kenya and spread to Comoros and later to other islands of the Indian Ocean including La Reunion. *Ae. albopictus* was reported as the predominant species in La Reunion where the typical CHIKV vector, *Ae. aegypti*, is very scarce [5]. The emergence of CHIKV in La Reunion was associated with a single nucleotide change resulting in an alanine (Ala) to a valine (Val) substitution at E1 glycoprotein position 226 of an East-Central-South African (ECSA) genotype [6]. Experimental infections showed that the E1-226V variant infects midgut cells of *Ae. albopictus* more efficiently than the E1-226A variant. Moreover the E1-226V variant was better disseminated and transmitted by *Ae. albopictus* [7], [8]. During 2005–2006, the selected variant E1-226V spread to the neighbouring islands in the Indian Ocean: Mayotte, Mauritius and Madagascar, where it was mainly transmitted by *Ae. albopictus* [6], [9], [10]. Later, phylogenetic analysis showed that the amino acid substitution E1-A226V in CHIKV had emerged almost simultaneously on three separate occasions: Indian Ocean, Central Africa, and Asia [11], [12]. Further studies showed that other substitutions in the viral genome, especially in the E1 and E2 genes, exert epistatic effects blocking the capacity of some genotypes to be better transmitted by *Ae. albopictus* via the E1-A226V substitution [13], [14]. In La Reunion, adaptation of CHIKV to *Ae. albopictus* was found to be associated with a fitness cost to the mosquito; *Ae. albopictus* died earlier than *Ae. aegypti* when infected with CHIKV E1-226V [15]. However, this increased mortality was not sufficiently rapid to interrupt transmission, because the extrinsic incubation period (time between feeding upon a viremic host and the presence of the virus in the saliva), is short. Indeed, both mosquito species were able to transmit the virus as soon as 2 days after CHIKV infection [16].

Here, we report a study to elucidate the competitive interaction between the original ECSA genotype E1-226A and the newly emerged variant E1-226V that arose following the shift from *Ae. aegypti* to *Ae. albopictus* as the dominant mosquito vector in the Indian Ocean region. While the amino acid substitution in the E1 protein (E1-A226V) is directly responsible for a significant increase in infectivity, dissemination and transmission by *Ae. albopictus*, the underlying molecular mechanism leading to the selection of the E1-226V variant is still unknown. We used direct competition assays *in vivo* to dissect mechanisms underlying the selection of the E1-226V by *Ae. albopictus* in the Indian Ocean region. This method was previously used to study the E1-A226V substitution using other mosquito populations and infectious clones [7]. In our study, when both variants were provided in a same blood-meal at equal proportions, we found that in *Ae. albopictus*, the E1-226V variant better disseminates from the midgut to secondary organs (wings and salivary glands) and was more efficiently transmitted through the saliva. Conversely, the native E1-226A variant was slightly better disseminated and better transmitted by *Ae. aegypti*. Interestingly, when both variants were inoculated at equal titers into mosquitoes, E1-226V was no longer favored for dissemination and transmission in *Ae. albopictus*. Because of the high mutation rate of arbovirus RNA genomes, the substitution E1-A226V is likely to occur at the same rate in *Ae. albopictus* and *Ae. aegypti*. To complete previous studies that pinpointed the role of a single substitution in an enhanced transmissibility of CHIKV by *Ae. albopictus* [7], [8], our results showed that the midgut barrier favors the dissemination of the E1-226V variant in *Ae. albopictus* and could explain why the E1-226V variant could have rapidly emerged as soon as the vector *Ae. albopictus* was present. Our finding corroborates previous results obtained using CHIKV infectious clones and laboratory colonies of *Ae. albopictus* [17], and presents detailed experiments using longitudinal sampling to compare infection outcome in different mosquito tissues and saliva.

## Materials and Methods

### Ethics Statement

The Institut Pasteur animal facility has received accreditation from the French Ministry of Agriculture to perform experiments on live mice [see permit numbers at http://webcampus.pasteur.fr/jcms/c_97619/agrements-des-animaleries] in compliance with the French and European regulations on care and protection of the Laboratory Animals. This study was approved by the Institutional Animal Care and Use Committee (IACUC) at the Institut Pasteur.

### Viruses

Two CHIKV isolates from La Reunion provided by the French National Reference Center for Arboviruses were used: (i) strain CHIKV 05.115 (E1-226A) isolated in June 2005 from a 24-year-old female presenting classical CHIK symptoms; and (ii) strain CHIKV 06.21 (E1-226V) isolated in November 2005 from a new-born male presenting meningo-encephalitis symptoms. Both strains were isolated on *Ae. albopictus* C6/36 cells from human serum and viral stocks were produced following three passages on *Ae. albopictus* C6/36 cells then harvested and stored at −80°C. The consensus sequence of these strains differed only by a substitution of an alanine by a valine at position 226 of the E1 glycoprotein. Viral titer estimated by serial 10-fold dilutions on Vero cells was 10^9^ plaque forming units (pfu)/mL for both E1-226A and E1-226V.

In addition, biological clones E1-A and E1-V, each clone corresponding to a single virus isolate, were produced by plaque purification and amplification on Vero cells from the variants E1-226A and E1-226V respectively. Six-well plates containing confluent monolayers of Vero cells were infected. Cells were incubated for three days under an overlay consisting of Dulbecco's Modified Eagle medium (DMEM), 2% Fetal Bovine Serum (FBS), 1% penicillin/streptomycin (Invitrogen) and 1% agarose at 37°C. The lytic plaques were removed by suction using a pipette. Each agarose plug containing a single clone was dissolved overnight at +4°C in DMEM before being re-amplified in C6/36 cells. Both biological clones were produced at high titers: 10^8^ pfu/mL for the clone E1-A and 10^8.6^ pfu/mL for the clone E1-V.

### Mosquitoes

*Aedes aegypti* Petite-Terre (AAPT) from Mayotte collected in December 2006 and *Ae. albopictus* Providence (ALPROV) from La Reunion collected in March 2007 were used for experimental infections. The F12 and F6 generations were respectively used for AAPT and ALPROV. Eggs were immersed in water for 24 h for hatching and larvae were reared in pans containing one yeast tablet per liter of dechlorinated tap water. Adults were maintained at 28±1°C, 80% relative humidity and a light:dark cycle of 16 h∶8 h. A constant supply of 10% sucrose was provided to adults. Females were fed three times a week on mice (OF1 mice from Charles River laboratories, France). All experiments involving live vertebrates were performed in compliance with the French and European regulations and according to the Institut Pasteur guidelines for laboratory animal husbandry and care.

### Experimental infections of mosquitoes

Infection assays were performed with one-week-old females in a BSL-3 laboratory. 60 individuals starved for 24 h prior to infection were placed in a plastic box. An average of four boxes was used per experiment.

#### Infectious blood-meals

Mosquitoes were allowed to feed for 15 min through a piece of pig intestine serving as the membrane covering the base of a glass feeder containing the infectious meal maintained at 37°C. The infectious blood-meal was composed of 1 mL of viral suspension and 2 mL of washed rabbit erythrocytes isolated from arterial blood collected 24 h before infection. Adenosine triphosphate was added at a final concentration of 5×10^−3^ M as a phagostimulant. Three types of blood-meals were provided: (i) one containing a viral suspension with both viruses E1-226A and E1-226V given at the same final titer (10^6.5^ pfu/mL for each virus), (ii) one containing a 1∶9 mix (E1-V:E1-A), and (iii) one containing a mix 9∶1 (E1-V:E1-A). The entire feeding period lasted less than one hour without any significant variation detected in viral titers. After feeding, engorged females were sorted on ice and placed in cardboard containers. They were maintained with 10% sucrose at 28±1°C, 80% relative humidity and a light:dark cycle of 16 h∶8 h.

#### Intrathoracic inoculations of mosquitoes

One-week-old females were inoculated with the two viruses, E1-226A and E1-226V, given at equal titers using the protocol described by Rosen and Gubler [18]. Each mosquito received 170 nL of viral suspension corresponding to 10^4.2^ pfu of each virus.

### Indirect Immunofluorescence assays (IFA)

To visualize the virus within the mosquito, different tissues were examined for the presence of viral antigens. Females were dissected in PBS 1X at days 3 and 7 post infection (pi) to isolate midguts and salivary glands. Organs were fixed with 4% paraformaldehyde for 15 min at room temperature (RT). Samples were rinsed once with PBS 1X. Midguts were incubated for 90 min in PBS 1X containing 0.2% Triton X-100 at RT. Salivary glands were incubated for 45 min in PBS 1X containing 0.1% Triton X-100. After two washes in PBS 1X, samples were incubated with 0.1% Tween (in PBS 1X and 1% bovine serum albumin (BSA)) for 30 min for salivary glands or 60 min for midguts at RT. Samples were incubated with mouse anti-CHIKV 3E4 antibodies specific to E2 glycoprotein (kindly provided by Dr. P. Desprès, Institut Pasteur) diluted at 1∶100 in PBS 1X with 1% BSA, for 1 h at RT for salivary glands or overnight at 4°C for midguts. Samples were washed in PBS 1X three times and incubated with Alexa Fluor 555 conjugated goat anti-mouse immunoglobulin G (Invitrogen) (diluted 1∶400 in PBS 1X with 1% BSA). After 45 min, samples were washed as described above and incubated 30 min at RT with Alexa Fluor 488 conjugated Phalloidin (Invitrogen) (in PBS 1X and 1% BSA). Samples were washed as described above and then mounted with 10 µL of Prolong gold antifade reagent with DAPI (Invitrogen). Samples were incubated overnight at RT and protected from light. Samples were examined using a LSM 700 confocal microscope (Carl Zeiss).

### Saliva collection

To evaluate transmission, females were chilled and their wings and legs were removed. The proboscis was inserted into a 20 µL tip filled with 10 µL of FBS. After 45 min, medium containing the saliva was expelled under pressure into a 0.5 mL tube containing 40 µL of DMEM. Samples were added to a monolayer of Vero cells to detect infectious particles by plaque assay.

### Clone isolation and viral titration

To measure infection and dissemination, females were sacrificed at different days pi. Organs (midgut, wings and salivary glands) dissected from mosquitoes, were used to estimate the total number of infectious particles and the proportion of E1-226V relative to E1-226A. Six-well plates containing confluent monolayer of Vero cells were infected with 10-fold dilutions of virus. Cells were incubated 3 days at 37°C and 5% CO_2_ under an overlay consisting of DMEM (1X) with 2% FBS, 1% L-Glutamine, 1% penicillin/streptomycin/amphotericin (Invitrogen) and 1% agarose. The lytic plaques were localized and removed by suction using a pipette. Each agarose plug that contained an individual clone was dissolved overnight at +4°C in 50 µL of DMEM. Viral envelope lysis was performed by adding 50 µL of 0.2% Nonidet P-40 dissolved in DMEM.

### RT-PCR amplification

To detect the E1-A226V substitution, a RT-PCR targeting the E1 gene was conducted using the Titan One Tube kit (Roche). The one-step RT-PCR reaction was performed in a volume of 50 µL containing 5 µL RNA template, 29.5 µL ddH_2_O, 1 µL dNTP (10 mM), 2.5 µL DTT (100 mM), 10 µL RT-PCR buffer (5X), 0.5 µL sense oligonucleotide (50 µM), 0.5 µL anti-sense oligonucleotide (50 µM), 0.5 µL RNase inhibitor (5 U/µL) and 0.5 µL Titan enzyme mix. Primers were selected in the E1 gene in a region comprising the position 226: sense CHIKV-E1F 5′-GCTAAGCTCCGCGTCCTTTA-3′ and anti-sense CHIKV-E1R 5′-CACACTTGCCTTTCTTGCTG-3′. The amplification program was performed as follows: reverse transcription at 50°C for 30 min, an inactivation of RT enzyme step at 94°C for 2 min, followed by 35 cycles of 94°C 15 s, 60°C 30 s, 72°C 1 min 30 s, and a final step at 72°C for 10 min. The size of the product was 595 bp. PCR products were purified using the NucleoFast kit (Macherey Nagel) as specified by the manufacturer.

### Sequencing

Sequencing was carried out using the ABI Prism BigDye Terminator Cycle Sequencing Ready Reaction kit version 3.1 (Applied Biosystems). The sequencing reaction was performed in a volume of 10 µL containing 1 µL PCR product template, 5.2 µL ddH_2_O, 2 µL sequencing buffer (5X), 1 µL sense oligonucleotide CHIKVE1-F (4 µM) and 0.8 µL ABI Prism solution version 3.1. The sequencing program was performed as follows: 96°C 1 min followed by 30 cycles of 96°C 10 s, 50°C 5 s, 60°C 4 min. Sequence chromatograms were obtained on automated sequence analyzer ABI3730XL (Applied Biosystems).

### Statistical analysis

All statistical analyses were performed using the Kruskall-Wallis test for comparisons of means and the Fisher's exact test for comparisons of proportions from the STATA software (StataCorp LP, Texas, USA). Tests were applied at each time-point separately.

## Results

### E1-226V is better transmitted by *Ae. albopictus*

After exposure to the infectious blood-meal containing equal proportions of E1-226A and E1-226V, saliva was collected and analyzed from 20–30 mosquitoes at days 3, 5 and 7 pi.

**Figure 1:**
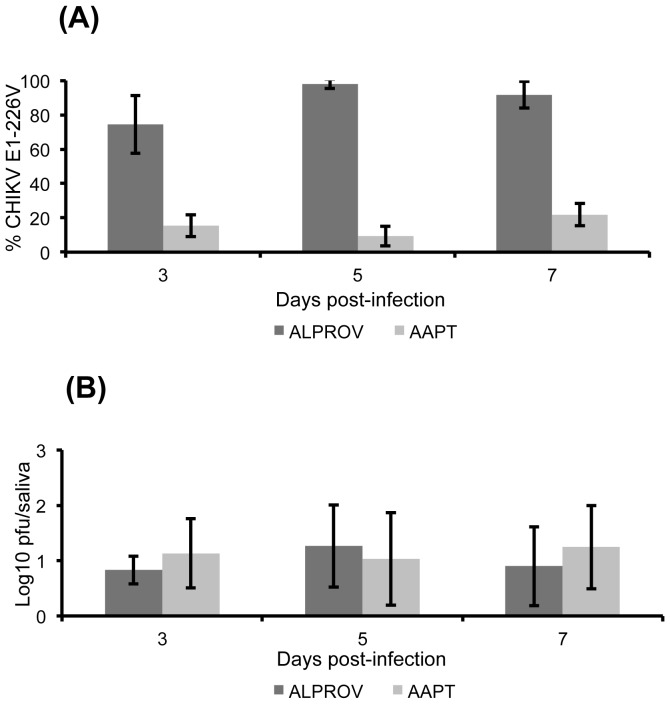
Effect of the E1-A226V substitution on CHIKV transmission in *Ae. aegypti* (AAPT) and *Ae. albopictus* (ALPROV). *Ae. aegypti* (light-grey) and *Ae. albopictus* (dark-grey) were orally infected with E1-226A and E1-226V provided at the same titer, 10^6.5^ pfu/mL. At days 3, 5 and 7 post-infection, mosquitoes were prepared for salivation. Collected saliva was used to inoculate monolayers of Vero cells. After 3 days at 37°C, clones were identified by sequencing. The proportion of E1-226V compared to E1-226A (A) and the number of infectious particles (B) were determined. Error bars show the confidence intervals (95%) for % CHIKV E1-226V and the standard deviations for Log10 pfu/midgut.

When examining proportions of E1-226V compared to E1-226A in saliva, ALPROV presented the highest proportions compared to AAPT (Fisher's exact test: p<0.05) (Figure 1A). For AAPT, mean proportions of E1-226V were low ranging from 15.5±6.4% at day 3 pi to 21.8±6.5% at day 7 pi. For ALPROV, values varied from 74.3±16.8% at day 3 pi to 91.7±7.8% at day 7 pi. Thus, ALPROV better transmits the E1-226V variant as measured in the saliva while the E1-226A variant is better transmitted in AAPT.

To define whether a differential replication capacity of the two variants occurred in the two mosquito species, the number of infectious viral particles excreted in saliva of AAPT and ALPROV was compared. The number of particles collected in saliva was similar in both mosquito species, regardless of the variant (Kruskal-Wallis test: p>0.05) and the day pi (Kruskal-Wallis test: p>0.05) (Figure 1B).

### E1-226V is better replicated and disseminated by *Ae. albopictus*

To determine if differential transmission of both variants was caused by differences in replication and dissemination capacities in mosquitoes, *Ae. aegypti* AAPT and *Ae. albopictus* ALPROV mosquitoes co-infected with E1-226A and E1-226V at equal proportions were sacrificed every day until day 7 pi to estimate viral infectivity and dissemination. Five mosquitoes dissected to isolate the midgut, wings and salivary glands were examined at each time point. The proportion of viral clones carrying the mutation E1-226V among examined clones was estimated. The number of infectious viral particles, regardless of the variant, excreted in saliva of AAPT and ALPROV was also compared.

#### CHIKV infectivity in mosquitoes after a blood-meal containing equal proportions of E1-226A and E1-226V

**Figure 2:**
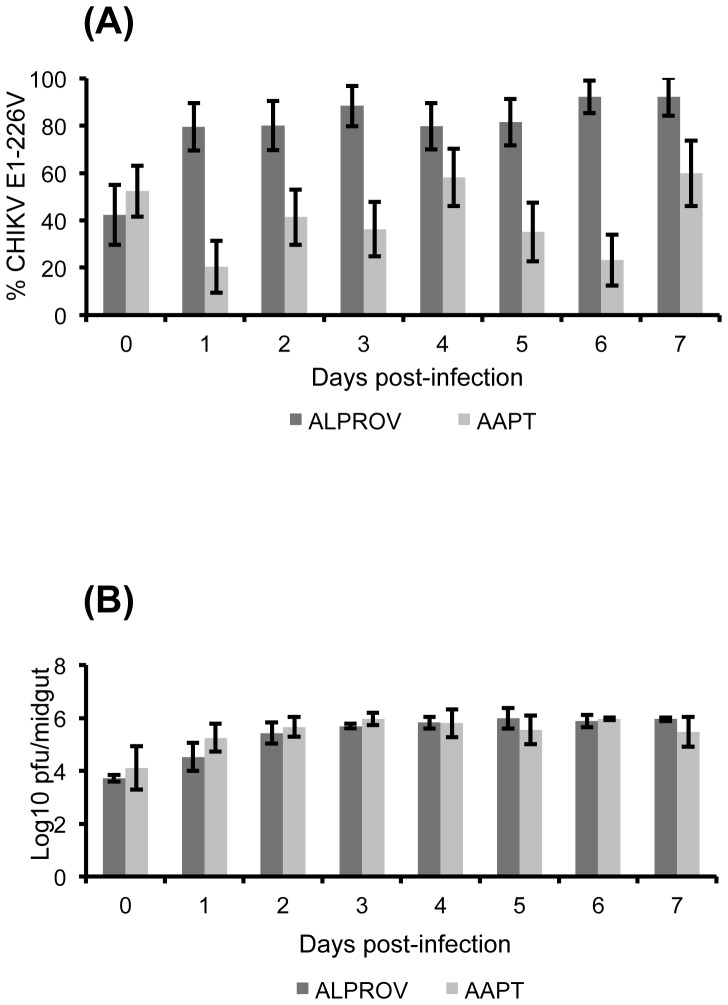
Effect of E1-A226V substitution on CHIKV infection in *Ae. aegypti* (AAPT) and *Ae. albopictus* (ALPROV). *Ae. aegypti* (light-grey) and *Ae. albopictus* (dark-grey) were orally infected with a blood-meal containing both E1-226A and E1-226V provided at the same titer, 10^6.5^ pfu/mL. Every day, 5 mosquitoes were sacrificed to isolate the midgut. The proportion of E1-226V compared to E1-226A (A) and the number of infectious particles (B) were determined. Error bars show the confidence intervals (95%) for % CHIKV E1-226V and the standard deviations for Log10 pfu/midgut.

When examining midguts, proportions of E1-226V were significantly different between AAPT and ALPROV from day 1 pi (Fisher's exact test: p<0.05) (Figure 2A). For AAPT, mean proportions of E1-226V were highly variable with values ranging from 20.4±11% at day 1 pi to 59.8±13.9% at day 7 pi. For ALPROV, mean proportions of E1-226V increased from 79.6±10.1% at day 1 pi to 92.2±8.0% at day 7 pi. This indicates that the E1-226V variant replicates more efficiently than E1-226A in *Ae. albopictus* midgut. When comparing proportions of E1-226V at a given day pi with the starting reference at day 0 pi for each mosquito species, significant increases were found from day 1 pi (Fisher's exact test: p<0.05) for ALPROV whereas differences were only detected at day 1 pi and day 6 pi (Fisher's exact test: p<0.05) for AAPT.

The number of infectious viral particles, regardless of the variant, was also compared in midguts between AAPT and ALPROV. No significant difference was found in midguts between the two species (Kruskal-Wallis test: p>0.05) (Figure 2B). This was confirmed by confocal microscopy, since CHIKV was detectable in midguts at days 3 and 7 pi in both species (Figure S1).

#### CHIKV dissemination in mosquitoes after a blood-meal containing equal proportions of E1-226A and E1-226V

**Figure 3:**
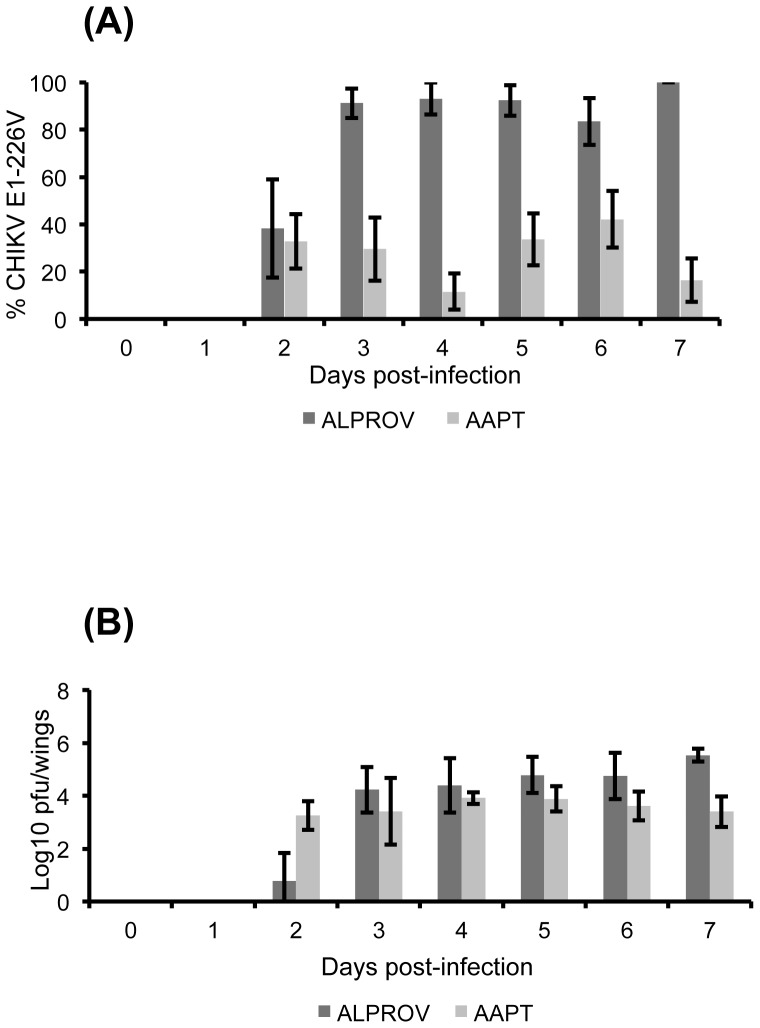
Effect of E1-A226V substitution on CHIKV dissemination to wings of *Ae. aegypti* (AAPT) and *Ae. albopictus* (ALPROV). *Ae. aegypti* (light-grey) and *Ae. albopictus* (dark-grey) were orally infected with a blood-meal containing both E1-226A and E1-226V provided at the same titer, 10^6.5^ pfu/mL. Every day, 5 mosquitoes were sacrificed to isolate the wings. The proportion of E1-226V compared to E1-226A (A) and the number of infectious particles (B) were determined. Error bars show the confidence intervals (95%) for % CHIKV E1-226V and the standard deviations for Log10 pfu/midgut.

**Figure 4:**
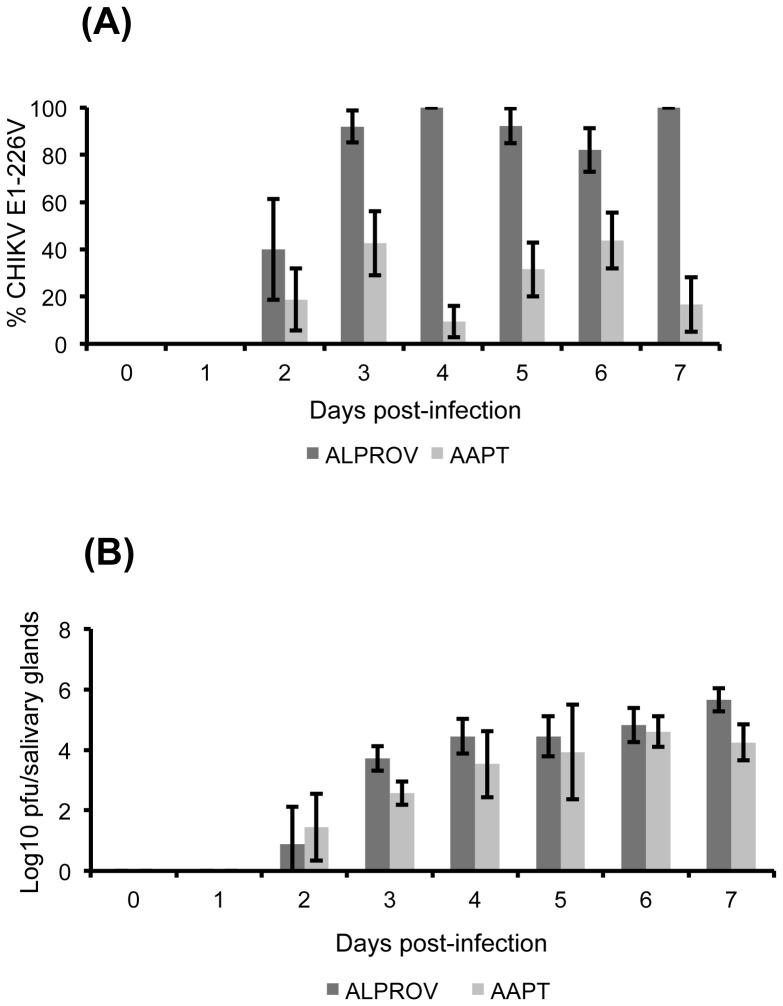
Effect of E1-A226V substitution on CHIKV dissemination to salivary glands of *Ae. aegypti* (AAPT) and *Ae. albopictus* (ALPROV). *Ae. aegypti* (light-grey) and *Ae. albopictus* (dark-grey) were orally infected with a blood-meal containing both E1-226A and E1-226V provided at the same titer, 10^6.5^ pfu/mL. Every day, 5 mosquitoes were sacrificed to isolate salivary glands. The proportion of E1-226V compared to E1-226A (A) and the number of infectious particles (B) were determined. Error bars show the confidence intervals (95%) for % CHIKV E1-226V and the standard deviations for Log10 pfu/midgut.

When analyzing wings, significant differences of E1-226V proportions compared to E1-226A were found between AAPT and ALPROV from day 2 pi when the virus started to be detected (Fisher's exact test: p<0.05) (Figure 3A). For AAPT, values were variable ranging from 32.8±11.5% at day 2 pi to 16.4±9.1% at day 7 pi. For ALPROV, mean proportions of E1-226V varied from 38.3±20.8% at day 2 pi to 100% at day 7 pi. In addition, when comparing the number of infectious viral particles, regardless of the variant, no significant differences were found between both species (Kruskal-Wallis test: p>0.05) (Figure 3B). When analyzing salivary glands, similar results were obtained (Figure 4A). For AAPT, proportions of E1-226V compared to E1-226A were very low and variable ranging from 18.7±13.1% at day 2 pi to 16.7±11.6% at day 7 pi. For ALPROV, mean proportions of E1-226V were higher than 80% from day 3 to day 7 pi. The five ALPROV mosquitoes tested at days 2, 4 and 7 pi have only ensured the dissemination of E1-226V variant. Thus this variant is preferentially disseminated in *Ae. albopictus*. On the other hand, the E1-226A variant preferentially disseminates in *Ae. aegypti* AAPT mosquitoes. When comparing the proportion of the E1-226A in the midgut and saliva of AAPT mosquitoes at day 3, 5 and 7 pi, the E1-226A variant was preferentially disseminated and transmitted in *Ae. aegypti* AAPT (Fisher's exact test: p<0.05).

When the number of infectious viral particles in salivary glands was compared between AAPT and ALPROV, regardless of the variant, no significant differences were found between both species (Kruskal-Wallis test: p>0.05) (Figure 4B). In both species, CHIKV was visualized by confocal microscopy in salivary glands at days 3 and 7 pi (Figure S1).

### *Ae. albopictus* preferentially transmits the biological clone E1-V

As we found that the E1-226V variant was preferentially selected for dissemination and transmission in *Ae. albopictus* ALPROV, we conducted a competition assay using unbalanced proportions of both variants to determine the threshold from which the E1-226V predominates in *Ae. albopictus*.

#### CHIKV dissemination in mosquitoes after a blood-meal containing unbalanced proportions of the biological clones E1-A and E1-V (9∶1 versus 1∶9)

**Figure 5:**
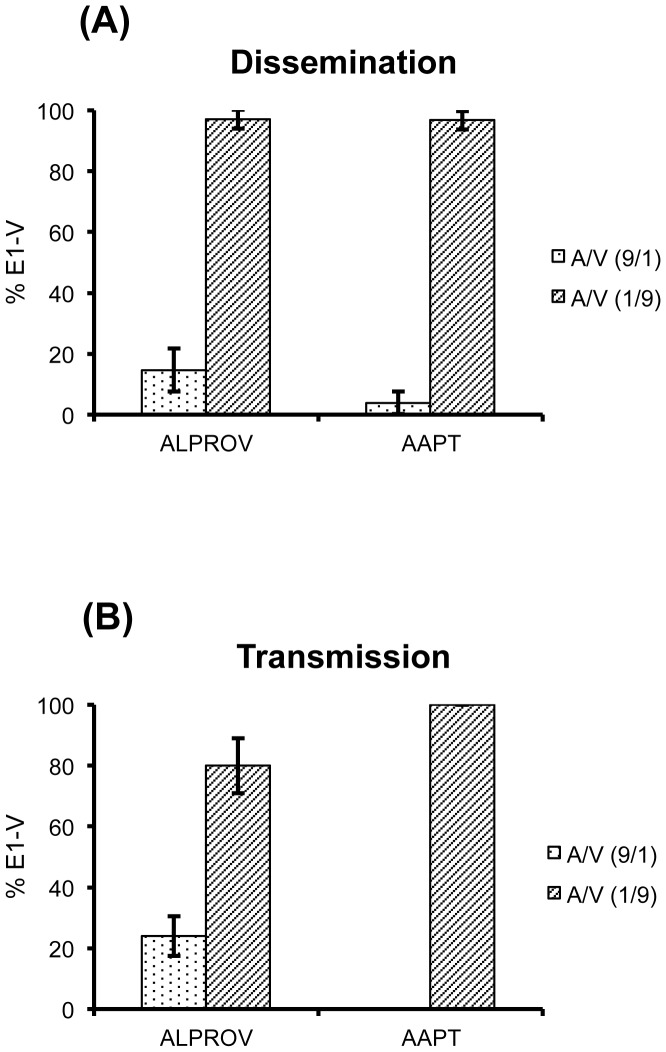
Unbalanced proportions of E1-226V and E1-226A provided in blood-meals to *Ae. aegypti* (AAPT) and *Ae. albopictus* (ALPROV). *Ae. aegypti* and *Ae. albopictus* were given an infectious blood-meal containing unbalanced proportions of two clones E1-A and E1-V isolated from the strains E1-226A and E1-226V, respectively. Two mixes were tested: one containing 1∶9 (E1-A:E1-V) and one containing a mix of 9∶1 (E1-A:E1-V). At day 7 post-infection, dissemination (A) and transmission (B) were examined by estimating the proportion of E1-V in wings and saliva, respectively, of five individual females. Error bars show the confidence intervals (95%).

After exposure to an infectious blood-meal containing different proportions of the biological clones E1-A and E1-V (9∶1 *versus* 1∶9), dissemination at day 7 pi was examined by estimating the proportion of clones carrying the E1-226V mutation in virus from the wings of ten individual females. In a blood-meal containing 9∶1 (E1-A/E1-V), mean proportion of the biological clone E1-V in wings was 3.8±3.7% for AAPT and 14.6±7.1% for ALPROV (Figure 5A). A significant difference of E1-V proportions was found between the two mosquito species at 9∶1 (E1-A/E1-V) (Fisher's exact test: p<0.05). When a blood-meal contained 1∶9 (E1-A/E1-V), mean proportion of E1-V in wings was 96.8±3.1% for AAPT and 97.2±3.2% for ALPROV. No significant difference of E1-V proportions was detected between AAPT and ALPROV at 1∶9 (E1-A/E1-V) (Fisher's exact test: p>0.05). In addition, significant differences were found between the two types of blood-meal for AAPT (Fisher's exact test: p<0.05) and ALPROV (Fisher's exact test: p<0.05).

#### CHIKV transmission in mosquitoes after a blood-meal containing unbalanced proportions of the biological clones E1-A and E1-V (9∶1 versus 1∶9)

Saliva of 30-40 mosquitoes infected orally with two different viral proportions, 9∶1 and 1∶9 (E1-A/E1-V) was examined at day 7 pi to assess transmission of the E1-V clone in AAPT and ALPROV. When the infectious blood-meal contained 9∶1 (E1-A/E1-V), mean proportion of E1-V in saliva was 0% for AAPT and 23.9±6.5% for ALPROV (Fisher's exact test: p<0.05) (Figure 5B). When the infectious blood-meal contained 1∶9 (E1-A/E1-V), mean proportion of E1-V was 100% for AAPT and 80.0±9.0% for ALPROV (Fisher's exact test: p<0.05). In addition, significant differences of E1-V proportions were found between both blood-meals for AAPT (Fisher's exact test: p<0.05) and ALPROV (Fisher's exact test: p<0.05).

These findings show that ALPROV mosquitoes were able to transmit a higher proportion of the biological clone E1-V after a blood-meal containing 9∶1 (E1-A/E1-V). Thus, after only one cycle of transmission in ALPROV mosquitoes, the proportion of E1-V has more than doubled, from 10% in the blood-meal to 24% in the saliva. For AAPT mosquitoes, no infectious particles of the biological clone E1-V were found in saliva under the same conditions.

### E1-226V is better transmitted after being preferentially selected in the *Ae. albopictus* midgut

To determine if infection of midgut is a key step in selecting the E1-226V variant in *Ae. albopictus*, we conducted two types of infection of AAPT and ALPROV with equal proportions of E1-226A and E1-226V: (i) by oral feeding of infectious blood-meals and (ii) by intrathoracic inoculation of mosquitoes. Proportions of E1-226V in wings and saliva were examined at day 7 pi to assess dissemination and transmission respectively.

#### Dissemination of CHIKV variants E1-226A and E1-226V in mosquitoes (oral feeding versus intrathoracic inoculation)

**Figure 6:**
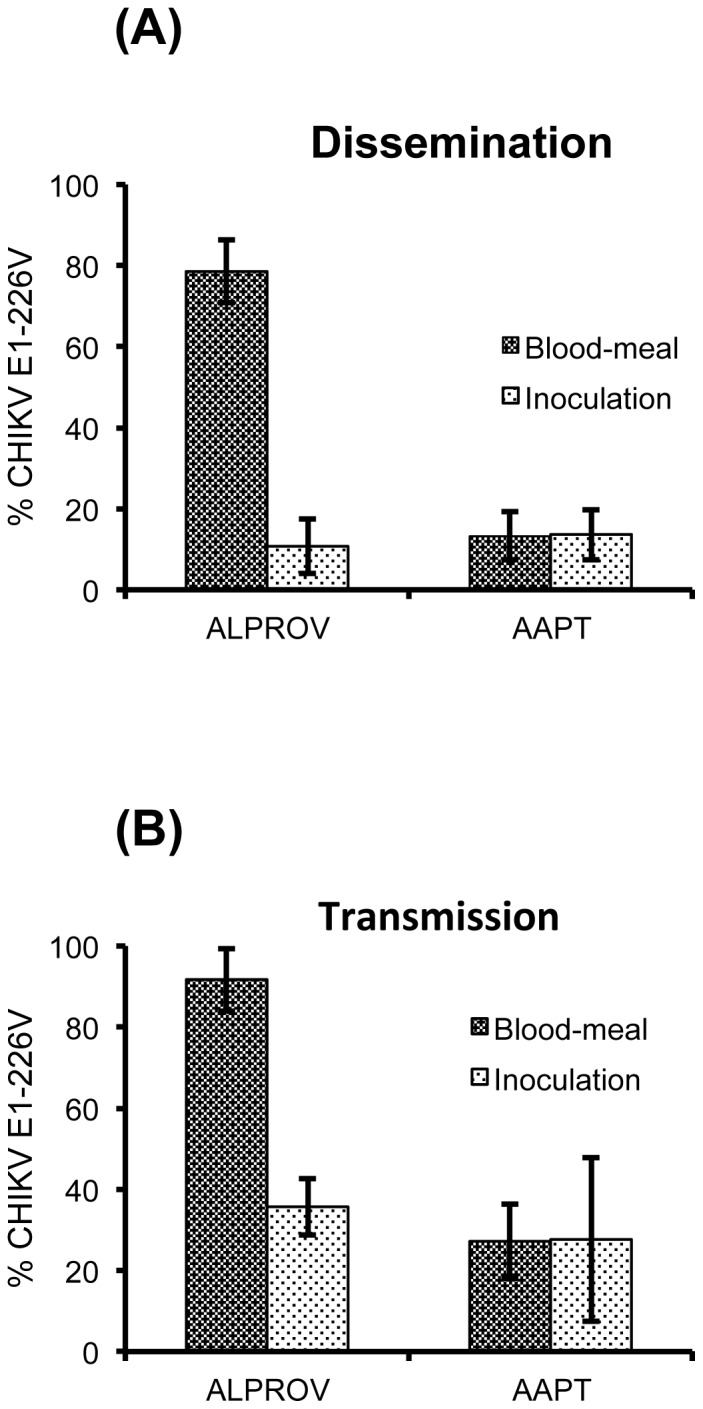
Effects of intrathoracic injection compared to blood-meal ingestion on CHIKV dissemination and transmission in *Ae. aegypti* (AAPT) and *Ae. albopictus* (ALPROV). *Ae. aegypti* and *Ae. albopictus* were orally infected or inoculated with E1-226A and E1-226V provided at the same titer, 10^6.5^ pfu/mL. At day 7 post-infection, dissemination (A) and transmission (B) were examined by estimating the proportion of E1-226V in wings and saliva. Error bars show the confidence intervals (95%).

When comparing proportions of E1-226V compared to E1-226A in wings of ten individual females, significant differences were found in ALPROV between individuals infected by ingestion of an infectious blood-meal and by intrathoracic inoculation (Fisher's exact test: p<0.05). Conversely, no significant difference was found for AAPT (Fisher's exact test: p>0.05) (Figure 6A). For ALPROV, mean proportions of E1-226V in wings were 78.5±7.8% when infection was performed by ingestion of infectious blood-meal and 10.8±6.2% by intrathoracic inoculation. For AAPT, mean proportions of E1-226V were 13.3±6.8% and 13.6±6.2%, respectively.

#### Transmission of CHIKV variants E1-226A and E1-226V in mosquitoes (oral feeding versus intrathoracic inoculation)

When comparing proportions of E1-226V in saliva of 30–40 mosquitoes, significant differences were found in ALPROV between individuals infected by ingestion of an infectious blood-meal and by intrathoracic inoculation (Fisher's exact test: p<0.05). For AAPT, no significant difference was found whichever infection method was used (Fisher's exact test: p>0.05) (Figure 6B). For ALPROV, mean proportions of E1-226V in saliva were 91.7±7.7% when infection was accomplished by ingestion of infectious blood-meal and 35.8±9.2% by intrathoracic inoculation. For AAPT, mean proportions of E1-226V were 27.2±7% and 27.6±20.1%, respectively.

These results show that midgut infection is essential for preferential dissemination and transmission of E1-226V in *Ae. albopictus*. When considering intrathoracic inoculations, we also observed that the E1-226A variant is more competitive for viral dissemination and transmission in both mosquito species.

## Discussion

The mosquito *Ae. aegypti* was the major mosquito vector in the initial phase of CHIKV emergence in Kenya [19] and Comoros [20] in 2004, whereas *Ae. albopictus* became the main vector in La Reunion [21] and Mayotte [10] during the acute phase of the outbreak. The shift from *Ae. aegypti* to *Ae. albopictus* was associated with an amino acid replacement in the E1 glycoprotein of the virus. This mutation was associated with efficient dissemination and transmission by *Ae. albopictus* [7], [8], hence leading to its selection in locations where *Ae. aegypti* was absent or rare. In this study, we showed that the selected E1-226V variant was no longer favored in *Ae. albopictus* when the two variants were provided at equal titers by intrathoracic inoculations into mosquitoes. This clearly demonstrates the key role of the midgut barrier in E1-226V dissemination through the mosquito body and subsequent transmission.

When CHIKV E1-226A and CHIKV E1-226V were provided in separate blood-meals, *Ae. aegypti* AAPT was found to be similarly susceptible to both viral strains [15]. By contrast, *Ae. albopictus* ALPROV was more susceptible to the E1-226V variant showing increased dissemination [15], [16]. Thus when provided alone, the E1-A226V substitution conferred a fitness advantage to the virus in *Ae. albopictus*. However, when E1-226A and E1-226V were provided together in a same blood-meal, there was no significant difference in viral infectivity and dissemination between AAPT and ALPROV as shown by indirect immunofluorescence assays on midguts and salivary glands. One explanation is that the variant E1-226V has diverted available cellular resources for its own replication. Indeed, when examining the proportion of each variant in tissues and organs, we found a fitness advantage of E1-226V in ALPROV. Once orally infected with a blood-meal containing both variants provided at same titers, the virus penetrates into the midgut epithelial cells and replicates. Virions produced in midguts were mainly the E1-226V variant in ALPROV. Later, virus released into the hemocele disseminates within the hemolymph and infects secondary tissues and organs. We found a higher proportion of E1-226V compared to E1-226A in ALPROV when analyzing wings and salivary glands, tissues indicative of viral dissemination. When examining the saliva released from mosquito salivary glands, more than 90% of viral clones detected in saliva were identified as E1-226V at day 7 pi in ALPROV. These results confirm previous data obtained using other *Ae. albopictus* populations and CHIKV infectious clones [7]. When co-infecting orally *Ae. aegypti* AAPT with both variants provided at same titers, no significant differences were found indicating that E1-226V selection occurs only in ALPROV. Nevertheless, a slight advantage for the E1-226A variant in *Ae. aegypti* AAPT was detected. When analyzing CHIKV dissemination and transmission in AAPT mosquitoes, E1-226V proportions ranged between 10% and 35%, suggesting an increased fitness of E1-226A. This may also explain why in regions where CHIKV was transmitted by *Ae. aegypti*, such as at the beginning of outbreaks in the Indian Ocean region, India and Singapore, the E1-226A variant was mainly isolated from human cases [22], [23].

To evaluate the magnitude of the advantage of the E1-A226V substitution in *Ae. albopictus*, both variants were provided at unbalanced proportions instead of at the same viral titers. ALPROV mosquitoes orally co-infected with a blood-meal containing 9∶1 (E1-A/E1-V) were able to transmit the variant E1-V at a higher proportion than would be expected. Indeed, seven days after infection, almost 24% of viral clones detected in saliva carried the E1-226V mutation. Experimental transmission by conducting several alternating passages of the virus between *Ae. albopictus* (through experimental infections using an artificial feeding system) and the mammalian host may favor the selection of E1-V increasing its dominance in ALPROV saliva. Conversely, no E1-V variants were detected in saliva of AAPT mosquitoes infected under the same conditions. Moreover, when orally co-infected with a blood-meal containing 1∶9 (E1-A/E1-V), AAPT and ALPROV mosquitoes maintained a high transmission of E1-V. Thus our findings confirmed that the E1-226V variant was strongly selected through enhanced transmission in *Ae. albopictus* ALPROV once ingested. Interestingly, replication capacity of both variants was similar. Indeed, when using *in vitro* systems (mosquito cells, *Ae. aegypti* Aag-2 and *Ae. albopictus* C6/36, and mammalian cells, Vero cells), no differences of growth curves based on RNA copies or infectious titers were detected (Figure S2). This result rules out a differential replication capacity of the two variants.

The substitution 226 is localized in the E1 envelope glycoprotein, which is responsible for fusion of viral and cellular membranes within the endosome [24]. When examining viral dissemination in ALPROV co-injected intrathoracically with E1-226V and E1-226A at the same titers, the E1-226V variant showed a significantly lower fitness loss compared to E1-226A. The passage of E1-226V through the midgut seems to be essential for its increased dissemination and subsequent transmission. The midgut barrier of insect vectors has been widely studied and seems to intervene in the attachment, penetration, replication and uncoating step of viral replication [25]. The role of the mosquito's midgut in the selection of new emerging variants has been shown for other arboviruses like West Nile virus [26] and Venezuelan equine encephalitis virus [27]. Our work demonstrated that for CHIKV, by bypassing the midgut barrier, E1-226V behaves similarly in *Ae. albopictus* and *Ae. aegypti*. Indeed, a significant decrease of E1-226V proportions in mosquito's wings and saliva was detected in *Ae. albopictus* intrathoracically-infected compared to orally-infected mosquitoes. Interestingly, the E1-226A variant was better disseminated and transmitted in *Ae. aegypti* whatever the way to infect (orally and intrathoracically). These results show that E1-226V selection in *Ae. albopictus* likely occurs during the process of midgut infection or during replication in midgut epithelial cells. When infectious clones presenting the E1-226A or E1-226V residues were separately inoculated into *Ae. albopictus*, similar profiles of viral replication were found [17]. When both infectious clones were provided intrathoracically at same titers, there was no advantage of the clone presenting the E1-226V residue [17].

For CHIKV, the molecular mechanism leading to the selection of the E1-226V variant is still unknown. Other mutations located close to the fusion loop, at the E1-86 residue and the E1-98 residue, may also affect virus entry [14]. Interactions between these residues may modulate the flexibility of the fusion loop and thus the dynamics between CHIKV and *Ae. albopictus* membranes during the phase of virus entry. Besides, it has been demonstrated that residues E2-60 and E2-211 in the E2 glycoprotein, may play an epistatic role that modulates the effect of the E1-A226V substitution [13]. Thus the E1-A226V substitution has improved the performance of the E1-226V variant, which colonizes mosquito internal organs more efficiently. Previous results showed that the E1-226V variant doesn't confer any advantage in *Ae. aegypti* mosquitoes [15]. One suggestion would be that a receptor present in *Ae. albopictus* midgut and not in *Ae. aegypti*, may play a significant role in interacting with the new CHIKV variant. Otherwise, the efficiency of E1-226V would be also found in *Ae. aegypti*.

RNA virus quasispecies facilitate rapid adaptation to changing host environments such as a shift in the vector species responsible for virus transmission to vertebrate hosts. The E1-A226V substitution in CHIKV that occurred in the spectrum of mutants was preferentially selected in *Ae. albopictus*. Interestingly, this particular adaptive substitution was speculated to have emerged independently at least three times during the last outbreaks [11], [12]. Our model based on mosquito strains colonized from field-collected mosquitoes and viral strains isolated from patients during the 2005–2006 outbreak in La Reunion, gives a plausible explanation on how the E1-226V variant emerged in the Indian Ocean region and strongly supports the scenario proposed by Tsetsarkin et al. [7]. The E1-226V variant in presence of other variants including the E1-226A was selected in response to a requirement for transmission by *Ae. albopictus*. Therefore, the predominance of *Ae. albopictus*, which transmits the variant E1-226V more efficiently after being selected in the mosquito midgut, is likely to be the main component leading to the explosive CHIKV outbreaks in the islands of the Indian Ocean. These findings bring new insight into the role of *Ae. albopictus* in contributing to the expansion of emerging arboviruses. *Ae. albopictus* is becoming widespread in Europe and other northerly regions, opening up the possibility of invasion of temperate countries by other tropical arboviruses.

## Supporting Information

Figure S1 **Histological localization of CHIKV in** ***Ae. aegypti*** **(AAPT) and** ***Ae. albopictus*** **(ALPROV).** Mosquitoes were orally infected with both viruses provided at a same titer, 10^6.5^ pfu/mL. At days 3 (B, D, G, I) and 7 (C, E, H, J) post-infection, mosquitoes were dissected to analyze midguts (A to E) and salivary glands (F to J). The nucleus was labeled using DAPI (blue), actin network with Alexa 488 phalloidin (green), and CHIKV using a monoclonal mouse anti-CHIKV antiboby and Alexa 555 goat anti-mouse antibody (red). The magnification was 25X.
(DOC)

Figure S2 **Growth curves of CHIKV, E1-226A and E1-226V, in three cell lines.** (A) *Ae. aegypti* Aag-2 cells, (B) *Ae. albopictus* C6/36 cells and (C) Green monkey Vero cells were infected with E1-226A (black) and E1-226V (grey) at a MOI of 0.1. After adsorption, the inoculum was removed and cells were washed. Then, medium with 2% FBS was added and cells were incubated at 28°C for mosquito cells and 37°C with 5% CO_2_ for mammalian cells. Supernatants were collected at 2, 4, 6, 8, 10, 24 and 72 hours post-infection (pi). The number of CHIKV genomes was determined by quantitative RT-PCR and the number of infectious viral particles by plaque titration of the same samples.
(DOC)
