# Supplementary material for: Dissemination and Transmission of the E1-226V Variant of Chikungunya Virus in Aedes albopictus Are Controlled at the Midgut Barrier Level
Source: PLoS One. 2013 Feb 21;8(2):e57548. doi: 10.1371/journal.pone.0057548 (PMC3578806; doi:10.1371/journal.pone.0057548)

**Figure S1. Histological localization of CHIKV in *Ae. aegypti* (AAPT) and *Ae. albopictus* (ALPROV).** Mosquitoes were orally infected with both viruses provided at a same titer, 10^6.5^ pfu/mL. At days 3 (B, D, G, I) and 7 (C, E, H, J) post-infection, mosquitoes were dissected to analyze midguts (A to E) and salivary glands (F to J). The nucleus was labeled using DAPI (blue), actin network with Alexa 488 phalloidin (green), and CHIKV using a monoclonal mouse anti-CHIKV antiboby and Alexa 555 goat anti-mouse antibody (red). The magnification was 25X.


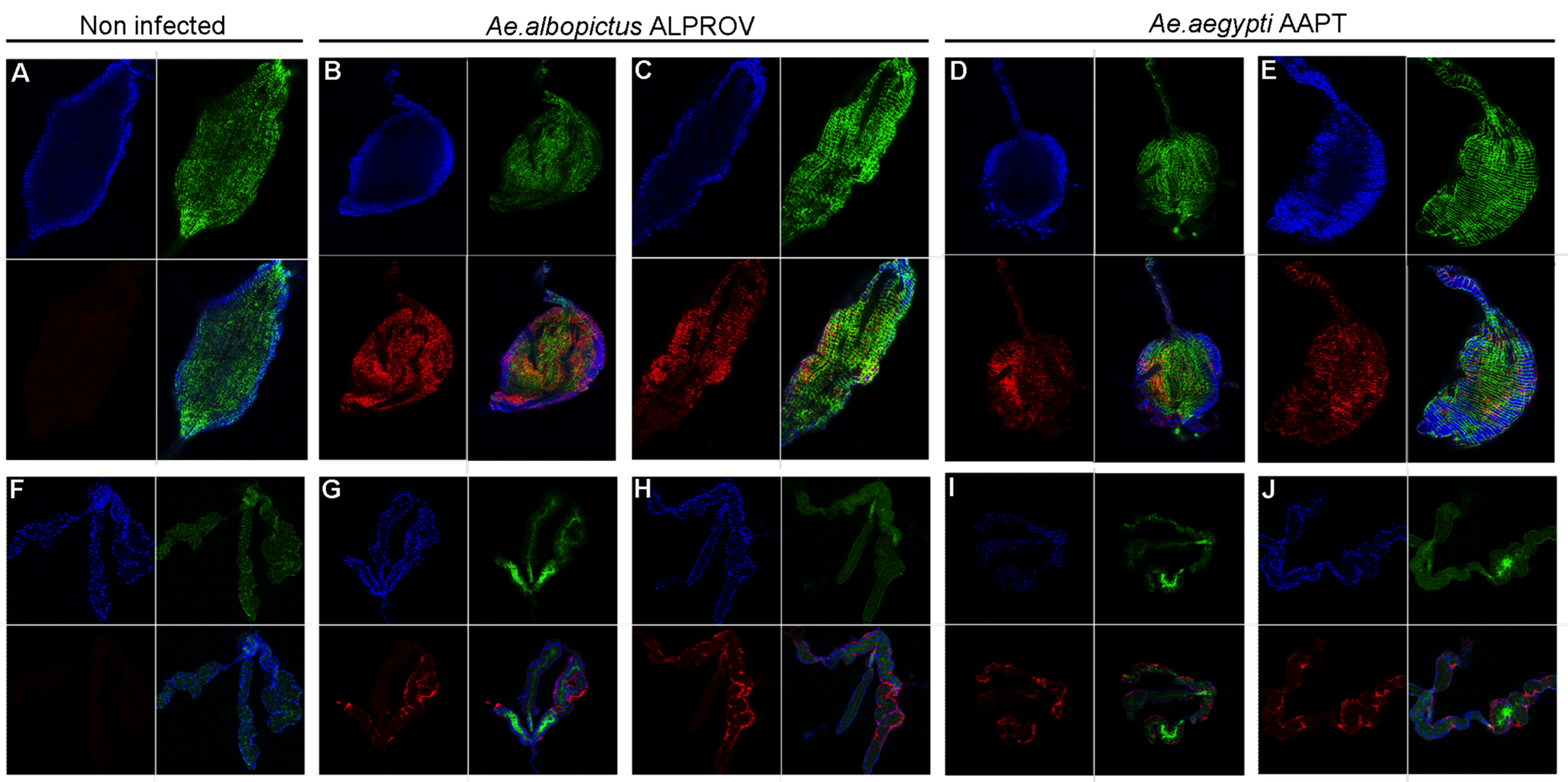

Supplement: Figure S1 — Histological localization of CHIKV in Ae. aegypti (AAPT) and Ae. albopictus (ALPROV). Mosquitoes were orally infected with both viruses provided at a same titer, 106.5 pfu/mL. At days 3 (B, D, G, I) and 7 (C, E, H, J) post-infection, mosquitoes were dissected to analyze midguts (A to E) and salivary glands (F to J). The nucleus was labeled using DAPI (blue), actin network with Alexa 488 phalloidin (green), and CHIKV using a monoclonal mouse anti-CHIKV antiboby and Alexa 555 goat anti-mouse antibody (red). The magnification was 25X. (DOC) [file pone.0057548.s001.doc]
