# Supplementary material for: Dissemination and Transmission of the E1-226V Variant of Chikungunya Virus in Aedes albopictus Are Controlled at the Midgut Barrier Level
Source: PLoS One. 2013 Feb 21;8(2):e57548. doi: 10.1371/journal.pone.0057548 (PMC3578806; doi:10.1371/journal.pone.0057548)

**Figure S2. Growth curves of CHIKV, E1-226A and E1-226V, in three cell lines.** (A) *Ae. aegypti* Aag-2 cells, (B) *Ae. albopictus* C6/36 cells and (C) Green monkey Vero cells were infected with E1-226A (black) and E1-226V (grey) at a MOI of 0.1. After adsorption, the inoculum was removed and cells were washed. Then, medium with 2% FBS was added and cells were incubated at 28°C for mosquito cells and 37°C with 5% CO_2_ for mammalian cells. Supernatants were collected at 2, 4, 6, 8, 10, 24 and 72 hours post-infection (pi). The number of CHIKV genomes was determined by quantitative RT-PCR and the number of infectious viral particles by plaque titration of the same samples.


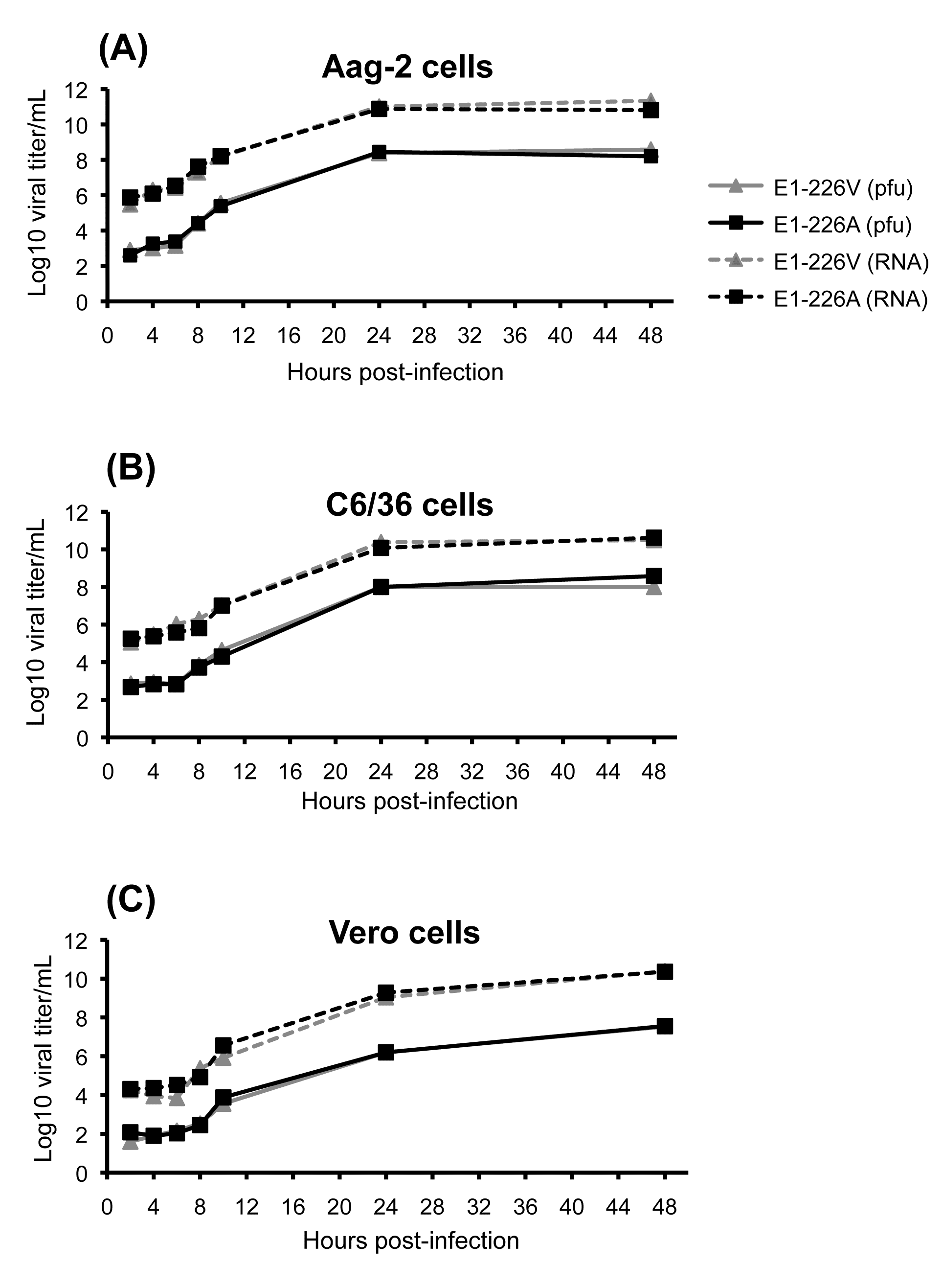

Supplement: Figure S2 — Growth curves of CHIKV, E1-226A and E1-226V, in three cell lines. (A) Ae. aegypti Aag-2 cells, (B) Ae. albopictus C6/36 cells and (C) Green monkey Vero cells were infected with E1-226A (black) and E1-226V (grey) at a MOI of 0.1. After adsorption, the inoculum was removed and cells were washed. Then, medium with 2% FBS was added and cells were incubated at 28°C for mosquito cells and 37°C with 5% CO2 for mammalian cells. Supernatants were collected at 2, 4, 6, 8, 10, 24 and 72 hours post-infection (pi). The number of CHIKV genomes was determined by quantitative RT-PCR and the number of infectious viral particles by plaque titration of the same samples. (DOC) [file pone.0057548.s002.doc]
